# A Review of Radiomics in Predicting Therapeutic Response in Colorectal Liver Metastases: From Traditional to Artificial Intelligence Techniques

**DOI:** 10.3390/healthcare10102075

**Published:** 2022-10-19

**Authors:** Fatma Alshohoumi, Abdullah Al-Hamdani, Rachid Hedjam, AbdulRahman AlAbdulsalam, Adhari Al Zaabi

**Affiliations:** 1 Department of Computer Science, College of Science, Sultan Qaboos University, P.O. Box 36, Muscat 123, Oman; 2 Department of Human and Clinical Anatomy, College of Medicine & Health Sciences, Sultan Qaboos University, P.O. Box 36, Muscat 123, Oman

**Keywords:** radiomics, CT, texture features, colorectal cancer, liver metastases

## Abstract

An early evaluation of colorectal cancer liver metastasis (CRCLM) is crucial in determining treatment options that ultimately affect patient survival rates and outcomes. Radiomics (quantitative imaging features) have recently gained popularity in diagnostic and therapeutic strategies. Despite this, radiomics faces many challenges and limitations. This study sheds light on these limitations by reviewing the studies that used radiomics to predict therapeutic response in CRCLM. Despite radiomics’ potential to enhance clinical decision-making, it lacks standardization. According to the results of this study, the instability of radiomics quantification is caused by changes in CT scan parameters used to obtain CT scans, lesion segmentation methods used for contouring liver metastases, feature extraction methods, and dataset size used for experimentation and validation. Accordingly, the study recommends combining radiomics with deep learning to improve prediction accuracy.

## 1. Introduction

Colorectal cancer (CRC) is one of the most common types of cancer [1]. Colorectal cancer (also known as bowel cancer) is the third most common cancer in the world. It is the third most common cancer among men and the second most common cancer among women. In 2020, there were more than 1.9 million new cases of colorectal cancer [2]. As a result of pre-existing pre-cancerous colon polyps (adenomatous polyps), colon cancer may develop over 5 to 20 years [3,4,5]. Approximately 135,430 new cases of colorectal cancer were diagnosed in the United States in 2017, resulting in 50,260 deaths [6]. Additionally, CRC is the second most common cause of cancer-related death among men and women, with approximately 52,020 deaths reported in 2019 in the US [7,8]. 

Several factors contribute to the increased incidence of CRC globally, including low- fiber and high-fat diets, excessive consumption of red meat, and sedentary lifestyles [9]. In addition, CRC is often detected at an advanced metastatic stage due to late detection of the tumor due to a lack of or poor adherence to screening programs [4].

CRC is classified into four stages based on the extent to which the tumor has spread from the colon to lymph nodes and distant organs [10]. A screening program for CRC has resulted in the early detection and removal of premalignant polyps or early-stage cancers, thereby improving survival rates [3]. Nonetheless, 20–25% of cases present with distant metastatic disease at diagnosis [11,12,13]. In addition, there are almost 20–40% of late-stage CRC patients present with liver metastasis [14,15], which has been explained through the hematogenous spread of cancerous cells throzugh the portal veins [14,16,17,18,19]. During the advanced stage of CRC, the survival rate is inferior, and the treatment options are minimal [20]. However, the high accuracy of CRC detection and the effectiveness of early detection treatment will assist in reducing the incidence rate before the transformation of benign polyps to malignant tumors [21]. A patient’s prognosis is greatly affected by their liver metastases, and the average survival time for patients with hepatic metastases from gastrointestinal cancers is only 6 months without appropriate treatment. Therefore, it is essential to accurately predict and differentiate liver metastases from CRC to make a proper therapeutic plan and improve the patient’s prognosis [22].

The work-up of CRC patients involves the use of radiological imaging methods, including colonoscopy, computerized tomography (CT scan), magnetic resonance imaging (MRI), ultrasound, chest x-ray, and positron emission tomography (PET) or PET-CT scan [23]. Computed tomography (CT) is the most widely used imaging technology for the staging of CRC [24]. Depending on the stage of the disease, patients may receive surgery, chemotherapy, and radiotherapy [16]. The more advanced the disease, the fewer treatment options are available, and most patients are treated aggressively or remain on palliative care. It is worth mentioning here that CRC patients with unresectable liver metastases usually receive systemic chemotherapy before or after surgery [7,16]. Based on the size of the metastatic liver lesions, the response to chemotherapy is assessed, and subsequent treatment decisions are made. However, the response of each patient to chemotherapy alone or combined with targeted therapy differs, and the benefits are fewer. 

Consequently, assessing the response to these therapies is imperative to avoid their toxic effects and high costs [25]. There is a strong correlation between the therapeutic response of liver metastases and their prognosis [14]. The Response Evaluation Criteria in Solid Tumors (RECIST 1.1) is commonly used to assess the response to treatment. It measures the difference between the longest axial diameter of the metastatic liver lesion before and after chemotherapy [12,15,16]. Tumors are categorized as responsive (if their size decreases), non-responsive (if their size does not change), and progressing (if their size increases). However, RECIST1.1 is limited because it does not consider the spatial heterogeneity of metastatic lesions [16,26]. RECIST does not accurately predict the response to bevacizumab in patients with CRC liver metastases, according to a recent study [27]. This is due to the fact that bevacizumab’s cytostatic action has a small impact on liver tumor size. Patients with respectable CRC liver metastases treated with bevacizumab plus XELOX (capecitabine and oxaliplatin) were found to respond better to CT morphological criteria than RECIST [27]. In cancer treatment, tumor morphology is considered a biomarker. While it is a useful biomarker, it is not a robust one since it is unable to predict the response to treatment in patients receiving systemic therapy [28]. Additionally, different morphological parameters have been reported by different studies, but none have been confirmed by all authors. Additionally, morphological criteria (e.g., tumor size) can be modified by chemotherapy, and it is unclear which value should be taken into consideration (prior to or after treatment). Furthermore, tumor morphology provides only a snapshot of the tumor and does not reflect its evolution over time [28].

Recently, radiomics-based approaches have gained attention due to their high prediction power for response to chemotherapy in various types of tumors, including liver metastases [11,12,16,19,26,29,30,31,32,33,34]. CT texture analysis is useful for diagnosing, staging, and assessing therapy response in several studies [12,14,16,35]. Moreover, imaging techniques can potentially characterize the histopathological features of CRCLM [36]. For example, the feature of the interface between the normal liver and tumor influences the chemotherapy selection [36]. However, there is an absence of studies that provide robust validation of imaging techniques [36].

Nonetheless, several challenges hinder the clinical application of radiomics feature analysis, beginning with variations in medical image acquisition protocols and moving on to standardizing radiological scores. Therefore, an examination of recent studies focusing on the use of CT texture features to predict the therapeutic response to CRC with liver metastases is presented in this review. The primary objective of this study is to explore the standard CT texture features for therapeutic response assessment in CRC with liver metastases, the CT acquisition parameters, existing radiomics texture feature extraction tools, and the common limitations that can be addressed in future research. 

**Figure 1:**
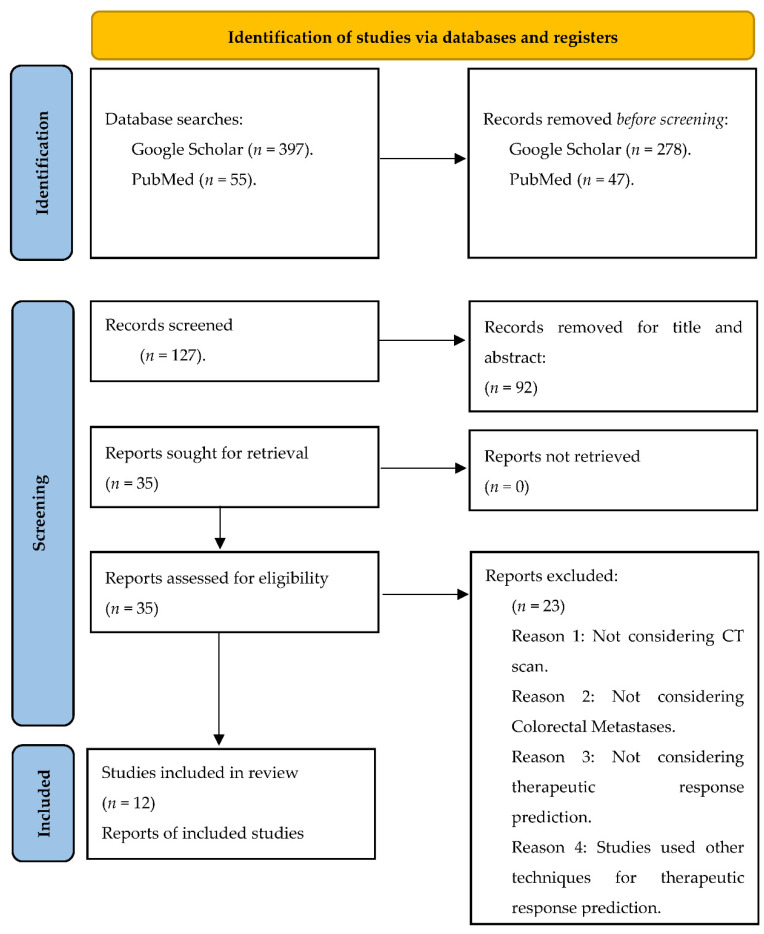
An illustration of the PRISMA flow diagram for a systematic review, including the database searches, abstract screening, and full text retrieval.

As part of this review, various online resources regarding CRC liver metastases and radiomics of CT were collected from different scientific journals. In this review, we reviewed studies related to CRC liver metastases and the radiomics of CT for predicting therapeutic response. A methodology diagram in Figure 1 outlines the criteria for including and excluding studies from the review.

As shown in Figure 1, only 12 studies have utilized radiomics of CT to predict the response to chemotherapy in patients with CRC liver metastases.

The rest of the paper is structured as follows: A brief overview of radiomics’ role in predicting therapeutic response to liver metastases in CRC is presented in Section 2. Then, several factors that influence radiomics, such as CT acquisition parameters, contouring methods of liver metastases, and extraction techniques of texture features, are described and compared in Section 3. Finally, the conclusion is drawn in Section 4.

## 2. Background

In both healthcare and computer science, radiomics is a promising field. For example, it plays a significant role in cancer diagnosis when applied to radiological imaging techniques such as CT, MRI, PET-scan, and others [37]. This approach uses radiological images to extract high-throughput quantitative features that can be used for diagnosing and assessing therapeutic outcomes [14,38]. 

Quantitative imaging features define the texture of tumors [39]. The quantitative imaging features include shape, intensity, volume, size, and texture, which provide detailed information on tumor microenvironment and phenotype compared to laboratory results, clinical reports, and genomic or proteomics analyses [22]. Additionally, radiomics measures spatial and temporal intramural heterogeneity non-invasively [40]. Thus, it is used to extract tumor features inaccessible to the naked eye [41]. Radiomics has provided significant results by identifying responders and non-responders across various treatment options, including surgery, chemotherapy, immunotherapy, and targeted therapy [38]. It is possible to predict the effectiveness of chemotherapy. Radiological tumor response has been associated with decreased entropy and increased homogeneity of liver lesions following chemotherapy. Some studies have reported that it is possible to predict response to systemic therapy by analyzing the images at the time of diagnosis prior to chemotherapy; higher entropy and lower homogeneity in liver metastases were associated with a higher response rate. As compared to the standard RECIST criteria, texture analysis provided a more accurate and earlier prediction [28].

It has been tested and applied to several types of cancer and has shown promising results [42]. In addition, using already available radiological images can extract such features non-invasively and with high predictive power [6].

**Figure 2:**
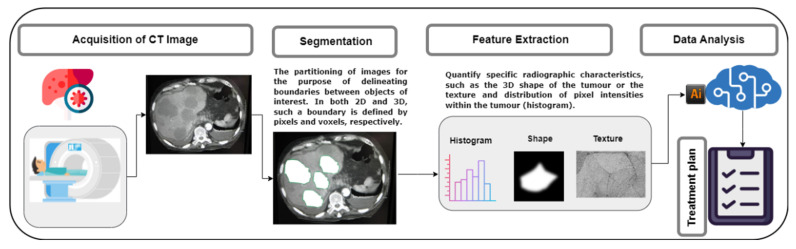
Radiomics workflow.

Figure 2 illustrates the radiomics workflow: 

According to Figure 2, a radiomics workflow is composed of four main steps: (1) acquisition of high-quality and standardized imaging; (2) segmentation of the region of interest (ROI), which can either be done automatically or manually by an experienced radiologist or radiation oncologist; (3) extraction of quantitative features from the segmented (ROI); and (4) analysis of the extracted features [39,43]. 

CT scans, as part of CRC patients’ follow-up, are widely used to detect liver metastases [6,44]. CT texture analysis is a mathematical approach for quantifying cancer heterogeneity by using algorithms that calculate features found in the ROI, such as coarseness distribution, irregularity of pixel intensities, and grey-level intensities [15,30]. As demonstrated in several studies, CT texture analysis can help diagnose, stage, assess therapy response, and identify disease survival biomarkers [16,45,46]. In addition, it helps predict chemotherapy responses and classify patients into two groups (responders and non-responders) [11,16]. As a result, there has been an increasing interest in using radiomics to diagnose cancers and predict their response to treatment [14,38].

Among the strengths of radiomics is the ability to provide early prediction of the outcome and noninvasive estimation of pathology specifics of colorectal metastases prior to collecting data normally collected only after surgery. Furthermore, the ability to interpret some radiomics features facilitates their implementation into clinical practice. For example, entropy and heterogeneity, especially after contrast enhancement, indicate active disease with heterogeneous clones, whereas homogeneity after chemotherapy indicates tumor necrosis [28]. In addition to exploring subtle changes in tumor and liver texture before and after treatment, radiomics can also be used to evaluate the response of CRLM lesions to chemotherapy [22]. To better understand the occurrence and development of diseases, radiogenomics can be used to discover radiomic features indicative of gene expression or polymorphism. Noninvasive and conventional imaging methods are used in radiogenomics to understand gene expression in diseases. At present, radiogenomics studies of liver tumors are limited [22].

## 3. Literature Review and Discussion

Many studies have been conducted to assess the therapeutic response of CRC patients with liver metastases [11,12,16,19,26,29,30,31,32,47,48,49]. According to the reviewed studies, the therapeutic response was assessed by analyzing different CT textures (features) extracted from CT scans of patients. The therapeutic response was evaluated after treatment with specific types of chemotherapy, such as bevacizumab-containing chemotherapy regimens [30], FOLFOX (5-FU, leucovorin, and oxaliplatin) or FOLFIRI (5-FU, leucovorin, and irinotecan) [11], regorafenib (a targeted cancer drug) [31], antiangiogenic therapies [11], and oxaliplatin chemotherapy [48]. The reviewed studies provide valuable information regarding radiomics’ role in predicting the therapeutic response to colorectal liver metastases. This work aims to review these studies, considering the common limitations in predicting the therapeutic response to colorectal liver metastases. This study reviewed 12 studies regarding the response of CRC liver metastases to therapy. It is worth mentioning that the assessment criteria used to assess the response to the treatment varies among studies. For example, among the four CT scanners included in [11], CT texture features such as skewness, mean attenuation, and standard deviation (SD) were compared. The authors of [12] calculated the ratio between texture features (T) of metastases and background liver (Metastases/Tliver) using entropy (E) and uniformity (U) extracted from texture features (T). A comparison was made between the texture features and clinical outcome parameters such as the extent of disease (number of metastases), response to chemotherapy, and overall survival. In [16], the authors evaluated the mean intensity (M), entropy (E), and uniformity (U) of the largest metastatic lesion using different filter values (0.0 ¼no/0.5¼fine/1.5¼medium/2.5¼coarse filtration). To evaluate the prediction of therapeutic response, ref. [48] used the least absolute shrinkage and selection operator regression models for the calculation of radiomic scores. 

The role of radiomics in determining the therapeutic response to chemotherapy has been confirmed in all studies. By using radiomics, biological data can be extracted from radiological images without invasive procedures, saving time, money, and eliminating any risk to the patient. In many tumors, radiomic analyses provide a precise assessment of biology, allowing for the identification of clinically relevant indices [14]. Using radiomics, liver lesions can be detected noninvasively. While traditional prognostic and predictive models have limitations, radiomic characteristics can be used to predict patient outcomes and treatment effectiveness. The field of radiomics has the potential to make a significant contribution to precision medicine. A study by Rao et al. in [16] demonstrated that radiomic features are superior to standard biomarkers for predicting chemotherapy response. In [11], radiomics was found to be useful in predicting the therapeutic response after cytotoxic chemotherapy in patients with colorectal cancer liver metastases. The authors concluded that a lower skewness on the 2D (two dimensional) image and a narrower standard deviation (SD) and a greater mean attenuation on the 3D image were significantly associated with an improved response to chemotherapy with FOLFOX or FOLFIRI for colon cancer hepatic metastases. According to [19], the radiomics signature outperformed known biomarkers (KRAS mutation status and tumor shrinkage based on RECIST 1.1) for predicting treatment sensitivity and for guiding decisions regarding the continuation of cetuximab treatment. As demonstrated in [26], it is possible to develop a radiomics model that can predict the likelihood of response of an individual metastasis in patients with colorectal cancer. In [30], the authors found that all texture parameters (radiomics), except kurtosis, changed significantly during treatment. According to their findings, radiomics may be useful for evaluating the efficacy of regorafenib treatment. Using FolFiri and bevacizumab as a first-line treatment for CRCLM, ref. [31] found that a radiomic signature (which represents a decrease in the sum of the target liver lesions (sTl), density, and computed texture analysis of the dominant liver lesion (Dll)) accurately predicts overall survival (OS) and identifies good responders more efficiently than RECIST 1.1 (Conventional evaluation criteria). Different CT texture features were calculated in each study. Thus, no standard cut-off values for specific texture features can be considered a stable feature for assessing good responders from poor responders to therapy. Several factors have contributed to these measurement variations, including using different CT scanners, inhomogeneity of the dataset, chemotherapy regimens, and segmentation methods for outlining the tumor region. A summary of the CT scan acquisition parameters used in the studies reviewed in this paper is shown in Table 1.

As illustrated in Table 1, various CT scan parameters have been used to acquire the CT scan, such as the scanner type (ranging from 4 slices up to 128 slices), tube voltage (100 to 120 kVp), radiation dose, or tube current (100 up to 242 mAs), slice thickness (2.5 up to 5mm), different scanner phases, and contrast (300 mg/mL up to 370 mg/mL). A recent study was conducted in 2019 to determine whether CT scan parameters affect the measurement of CT radiomics-based texture features of lung nodules [50]. In the study, it was found that CT scan parameters have a significant impact on the obtained imaging data. The study suggested normalization is required when the images are acquired with different CT scan parameters to analyze texture features accurately. Moreover, the study reported that other CT scan parameters could affect qualitative CT features due to the artifacts that affected the tumor texture [51]. As an example, noise increases with thinner slice thickness and vice versa. In [31], a thick slice was preferred over a thinner slice to minimize image noise’s influence on fine texture parameters. As reported in [52], slice thickness may affect measured radiomics features. However, according to Mackin et al. [53], some CT scan parameters have no significant effect on radiomics features.

Nonetheless, a significant difference was observed in extracted features when the scan tube current was between 30 mAs and 120 mAs, as reported in [51]. Therefore, it is recommended that high mAs be used to reduce motion artifacts [50]. Typically, CT scans are performed at high voltages (120–140 KVp). By increasing the KVP, a better image can be obtained, reducing scanning time. Furthermore, the kilovoltage setting affects dose and contrast. In [54] examined the effects of different iodine concentrations on the liver’s image quality of CT scans using a 128-slice scanner.

On the other hand, a higher iodine concentration (400 mg/mL) enhances the liver’s appearance in the portal phase and improves the overall quality of the image [54]. Numerous studies have demonstrated that higher iodine levels benefit CT scans [55,56]. Furthermore, using the same CT scan parameters facilitates the reproduction of radiomics features [51]. Obtaining a stable analysis of texture features from radiomics requires homogeneity in the dataset. In addition, all patients should receive the same treatment type and duration throughout the study. Furthermore, the segmentation and extraction techniques for texture features should be the same. As shown in Table 2, there are variations in the datasets, chemotherapy treatments, segmentation techniques, and feature extraction methods used in the reviewed studies.

As shown in Table 2, some reviewed studies used small datasets (ranging from 21 to 42) while others used large datasets (ranging from 230 to 667). Results obtained from a larger sample size are typically more stable than those obtained from a smaller dataset. Nevertheless, the small sample size is a common limitation in studies of CRC liver metastases using CT images, as reported recently [14]. In addition, the patient’s treatment regimens during these studies differed, as seen in Table 2. As a result, it is impossible to compare the measured features among these studies because the response differs from one therapy. Due to the high heterogeneity of CRC, patients respond differently to therapy for different metastatic lesions in the same patient [10,47]. Therefore, assessing the response in a tiny lesion leads to less accuracy. In CRC liver metastases, in the case of multiple lesions, different lesions show a similar response to chemotherapy [16]. Manual segmentation was the most commonly used segmentation method in the reviewed studies in which one or two readers (radiologists) manually delineate the region of interest. Indeed, the manual segmentation of ROI has an impact on texture features. Therefore, the inter-reader variability is critical and affects the extracted radiomics texture features. According to Rizzetto et al. in [57], describing the impact of inter-reader contouring variability on the textural radiomics of colorectal cancer liver metastases, segmenting liver metastases is a challenging procedure due to the location and boundaries of the liver. Consequently, 2D contouring has less effect on radiomics features than 3D contouring in terms of inter-reader variability [57]. As shown in Table 2, there is no standard tool for extracting texture features.

Most studies have used MATLAB and Python scripts (pyradiomics packages [58]). Some studies, however, used paid software such as TEXRAD, which is dedicated to scientific research, particularly in oncology. Radiologists and specialists developed it in IT healthcare to ensure the confidentiality of patient information. It extracts texture features from medical images, and over 100 academic publications rely on it. TEXRAD has been used in about eight studies related to colorectal cancer and texture features [8,44,45,59,60,61,62,63]. Some studies used free, open-source software to extract and quantify features. This software was written in Java and was intended for use by researchers, radiologists, and oncologists [64,65]. In the reviewed studies, there are variations in CT scan acquisition parameters, ROI segmentation methods, feature extraction tools, and even analysis methods used to predict response. The following, Table 3, summarizes the limitations of the reviewed studies:

The use of radiomics and radiogenomics in clinical medicine research is becoming increasingly popular due to their noninvasive nature and low cost. As a new field, it is still at an early stage, with numerous limitations. For example, most of the research data for radiomics comes from small samples and single center. In contrast, some data from multicenter are different due to different scanning equipment and scanning conditions. In addition, imaging delineation segmentation approaches may vary from one center to another or from one study to another [22]. Further, the results obtained by the studies require further validation and evaluation before they can be applied in clinical practice due to the instability of calculated texture features caused by variations in CT parameters, tumor segmentation, feature extraction techniques, and differences in treatment regimens’ patient status. Validation datasets are essential for improving generalizability of preliminary results [26]. Using a validation cohort of 90 patients, Ahn et al. demonstrated that certain texture features were independently correlated with the response to chemotherapy of the largest hepatic metastases. The filtered dataset shows prognostic correlations with survival, according to [30]. Further validation in larger prospective studies is, however, required. There are, however, some studies that did not incorporate validation into their models, such as [12,16]. In addition, studies produced contradictory results that were difficult to compare [41]. To achieve better outcomes in radiomics and radiogenomics, future research and development will need to address these issues. Because of technical complexity, data overfitting, lack of standardization for outcome validation, and unrecognizable confounding factors in databases, radiomics is still a relatively new field of study. However, by combining radiomics with other clinical information, correlation analysis can be performed with clinical results, and radiomics can thus provide countless imaging biomarkers for cancer diagnosis, detection, prognosis evaluation, prediction of treatment response, and disease monitoring [22].

Recent advances in radiomics and deep learning models, such as convolutional neural networks, have led to promising results, particularly in identifying and segmenting small lesions. As a result of deep learning (DL), automatic feature extractions are possible, reducing the laborious manual process of feature extraction. Combining deep learning with radiomics has shown remarkable results in recent studies such as [66]. A lack of studies, however, contributed to the use of deep learning for CRC liver metastases, as recently discussed in [14]. In addition, the evidence for using deep learning in conjunction with traditional hardcoded radiomics to predict chemotherapy response is still lacking [49]. In recent years, multimodal data fusion has become a significant research area due to its use in various applications in which multiple data sources are combined [67]. 

It is noteworthy that in the medical domain, the data is divided into non-image (patient’s medical records) and raw medical image data, which includes a large amount of undiscovered or hidden information. Thus, combining information from different modalities to improve diagnosis accuracy is challenging [68]. Data fusion combines data from various sources to obtain higher quality and more relevant information [69]. Essentially, this concept refers to integrating data from multiple sources to make accurate predictions [70]. Using data fusion methods has the primary goal of reducing detection inaccuracy probability and increasing reliability by combining data from various sources [69].

Furthermore, data from other sources can be used to observe the same features across multiple modalities, allowing for robust prediction [69,70]. It has been applied to various applications, including audio-visual speech recognition, emotion analysis, and medical image analysis [70,71]. Currently, multimodal applications have limitations in observing or learning the correlations among highly heterogeneous modalities [68,70]. At the final stage, the decision scores for each modality were combined without considering the inherent correlations between the modalities, such as image vs. non-image. Due to these limitations, multimodels suffer from low sensitivity and high specificity. In addition, integrating useful features across other modalities, such as hand-crafted features in the medical domain, requires robust domain knowledge, another limitation [67]. DL has recently been utilized to address these limitations by learning data representations and discovering correlations among features from multimodal datasets [68,72]. There is evidence from several studies that multimodal for DL achieves better results than unimodal, with the fusion of data from different modalities [68,69,73,74,75]. Based on their findings, DL models with fusion methods are promising in improving classification and prediction accuracy by combining different data sets with tight correlations and complementary information. However, the fusion methods vary between early, intermediate, and late fusion [67], indicating that it is still in its infancy in the medical domain. A lack of deep learning and multimodal methods for predicting therapeutic responses in CRCLM is shown in the following Table 4.

In the discussion, it became apparent that more studies are required to examine the use of advanced deep learning technologies combined with radiomics to evaluate therapeutic responses in patients with CRCLM. 

As a result, this study suggests investigating the use of the fusion method for predicting therapeutic response in patients with CRCLM by combining data from different sources (radiomics, clinical, and image).

## 4. Conclusions

Patients can avoid toxicity and adverse effects by predicting the response to chemotherapy early. Although radiomics plays an essential role in assessing the therapeutic response to chemotherapy, it still faces significant challenges in standardizing the CT acquisition parameters as well as automating the segmentation of liver metastases. A radiomics texture feature’s stability is crucial to assessing the therapeutic response to chemotherapy in CRC liver metastases. Therefore, further studies are required to evaluate texture features among different CT parameters and demonstrate a reasonable interpretation of the quantified features that can be applied as a standard approach in future studies. In addition, automatic lesion segmentation would facilitate radiomics analysis in clinical settings. According to the current review, deep learning can be used with radiomics to predict therapeutic responses to chemotherapy. Additionally, it suggests combining different data sources (CT, clinical data, and others) to improve prediction accuracy. 

## Figures and Tables

**Table 1:** Comparison between studies based on CT scan parameters.

Study Reference	CT Scan Acquisition Parameters					
	Scanner Type (Detector Rows)	Tube Voltage (kVp)	Radiation Dose (mAs)	Slice Thickness	Scanner Phase	Contrast
[29]	4 or 16 slices	NM	NM	NM	triphasic liver phase or single phase	Ioversol
[16]	16-slice or 64-slice	NM	NM	3 mm	PVP	NM
[11]	64-slice 16-slice 8-slice	120 kVp 120 kVp 120 kVp	200 mAs 200 mAs 250 mAs	3 mm 3 mm 2.5 mm	arterial and PVP phases PVP_ONLY	370 mgI/mL iopromide
[12]	64-slice	100 kVp–120 kVp	NM	3 mm or 5 mm	(PVP)	300 mgI/mL iopromide
[30]	256-slice	100 kVp or 120 kVp	100 mAs	5 mm	Non-contrast enhanced DCE-CT peak arterial enhancement DCE-CT (PVP)	320 mgI/mL Or 350 mgI/mL iodixanol
[31]	128-slice	120 kVp	210 mAs	5mm	NM	350 mgI/mL Iomeron
[32]	NM	NM	NM	3mm or 5mm	(PVP)	NM
[19]	NM	122 ± 6 kVp	242 ± 99 mAs	5.1 ± 1.0 mm	(PVP)	NM
[26]	NM	NM	NM	NM	(PVP)	NM
[47]	NM	NM	NM	NM	(PVP)	NM
[48]	iCT 256/IQon Spectral CT/Brilliance 64	NM	NM	3–5 mm	(PVP)	600 mgI/kg Iopamiron
[49]	Brilliance iCT	120 kVp	240–400 mAs	5 mm	(PVP)	100 mL Iopromide 370 mg/mL

Abbreviations: CT: computed tomography. kVp: kilovoltage peak (the peak potential applied to the X-ray tube). mAs: milliampere-second (tube current-time product). PVP-CT: portal venous phase computed tomography. NM: not mentioned.

**Table 2:** Comparison between studies based on different radiomics influencing factors.

Study	Dataset Size	Targeted Chemotherapy	Segmentation Method	Feature Extraction Tool	Extracted Features
[29]	50 patients	Chemotherapy and bevacizumab	NM	NM	Studies vary in measuring different radiomics features such as mean-intensity value, entropy, uniformity, histogram parameters, grey-level co-occurrence matrix, and other radiomics
[16]	21 patients	Capecitabine plus oxaliplatin (XELOX)	Manually	MATLAB Script	
[11]	145 patients	FOLFOX * FOLFIRI *	Manually	Medical Imaging Solution ^	
[12]	70 patients	Different regimens	Manually	In house-software written in Python (Pyradiomics package)	
[30]	27 patients	Bevacizumab and regorafenib	Intellispace 6.0 (ISP) ^^	TexRAD	
[31]	43 patients	FOLFOX ** FOLFIRI ** Alone or with bevacizumab	Manually	MATLAB Script	
[32]	230 patients	FOLFIRI * and bevacizumab)	Manually	TexRAD software	
[19]	667 patients	FOLFIRI * and cetuximab	Counters were drawn semi-automatically	MATLAB script	
[26]	24 patients	NM	Manual	NM	
[47]	24 patients	NM	Automatic	NM	
[48]	42 Patients	Oxaliplatin	Manual	3D slicer tool	
[49]	192 patents	oxaliplatin (CAPEOX or mFOLFOX6) or irinotecan (FOLFIRI or XELIRI)	Manual	Pyradiomics Package	

Abbreviation: NM: not mentioned. FOLFOX *: (5-FU, leucovorin, and oxaliplatin). FOLFIRI *: (5-fluorouracil, leucovorin, and irinotecan). FOLFOX **:(Oxaliplatin, 5-Fluorouracil and folinic acid). FOLFIRI ** (Irinotecan, 5-Fluorouracil and folinic acid). ^: For Segmentation and Texture Analysis (C++ BASED). ^^: Multimodality Tumor Tracking is a semi-quantitative 3D sculpt tool used for delineating tumors.

**Table 3:** Summary of limitations from the reviewed studies.

Main Common Limitations
Small dataset and data inconsistency: In most studies, external validation was required because they were retrospective studies conducted for a single institution. There was a difference in treatment among the patients.
Manual segmentation: One reader (without taking into account interobserver variation) performed an image segmentation. Subject bias. There is no standard method for determining the size of lesions.
More evaluation is required: The texture measurements were not retested to assess their repeatability. A single metastatic lesion was evaluated. It was only possible to extract features from the large lesion and not from all metastases. It is important to note that not all texture features were analyzed.

**Table 4:** Comparison among studies based on using deep learning and multimodal for predicting therapeutic response in CRCLM.

Study Reference	Radiomics Features	Machine Learning	Deep Learning	Multimodal
[29]	✗	✗	✗	✗
[16]	✓	✗	✗	✗
[11]	✓	✗	✗	✗
[12]	✓	✗	✗	✗
[30]	✓	✗	✗	✗
[31]	✓	✗	✗	✗
[32]	✓	✗	✗	✗
[19]	✓	✓	✗	✗
[26]	✓	✓	✗	✗
[47]	✓	✓	✗	✗
[48]	✓	✗	✗	✗
[49]	✓	✗	✓	✓

## Data Availability

Not applicable.
